# Time-dependent simulation of blood flow through an abdominal aorta with iliac arteries

**DOI:** 10.1007/s00249-024-01724-w

**Published:** 2024-10-18

**Authors:** Grzegorz Górski, Krzysztof Kucab

**Affiliations:** Institute of Physics, College of Natural Sciences, University of Rzeszów, ul. Pigonia 1, 35-310 Rzeszów, Poland

**Keywords:** Blood flow, Aortic stenosis, Turbulence models

## Abstract

Atherosclerosis is one of the important diseases of the circulatory system because atherosclerotic plaques cause significant disruption of blood flow. Therefore, it is very important to properly understand these processes and skillfully simulate blood flow. In our work, we consider blood flow through an abdominal aorta with iliac arteries, assuming that the right iliac artery is narrowed by an atherosclerotic lesion. Blood flow is simulated using the laminar, standard $k-\omega$ and standard $k-\epsilon$ models. The obtained results show that despite the use of identical initial conditions, the distribution of velocity flow and wall shear stress depends on the choice of flow simulation model. For the $k-\epsilon$ model, we obtain higher values of speed and wall shear stress on atherosclerotic plaque than in the other two models. The laminar and $k-\omega$ models predict larger areas where reverse blood flow occurs in the area behind the atherosclerotic lesion. This effect is associated with negative wall shear stress. These two models give very similar results. The results obtained by us, and those reported in the literature, indicate that $k-\omega$ model is the most suitable for blood flow analysis.

## Introduction

The rapid development of medicine requires progress in knowledge about the processes that occur in the human body. One of the tools used to expand this knowledge are numerical simulations, which allow one to visualize the behavior of specific organs of the human body and to predict health consequences. Numerical modeling of blood flow in 3D models with the use of Computational Fluid Dynamics (CFD) simulation allows one to obtain information about the distribution of hemodynamic parameters such as blood flow rate, flow velocity, pressure, or wall shear stress. Knowledge of these parameters is of great importance in the context of the appearance of pathological changes in a circulatory system, e.g. the formation and development of atherosclerosis or the development of aortic aneurysms. It is well known that regions with low shear stresses are prone to the formation of atherosclerosis (Ku et al. 28; Zarins et al. 59; Malek et al. 35; Tanweer et al. 50; Trenti et al. 54), whereas regions with high shear stresses induce protection against atherosclerosis (Malek et al. 35).

Arterial atherosclerotic disease is currently a very common disease that disrupts the normal structure and function of blood vessels. Therefore, the examination of blood flow disorders related to the appearance of arterial stenosis is one of the important topics related to this topic (Ku et al. 28; Zarins et al. 59; Bonert et al. 10; Taylor et al. 51; Malek et al. 35; Chakravarty and Sannigrahi 14; Siddiqui et al. 47; Rigatelli et al. 44; Owasit and Sriyab 40; Hussain et al. 22, 21; Lopes et al. 32; Alimohammadi et al. 3). Analytical solutions of a flow through aortic stenosis can only be obtained for its straight sections with the exponential, bell, or cosine shape of symmetric stenosis (Chakravarty and Sannigrahi 14; Mandal 36; Siddiqui et al. 47; Owasit and Sriyab 40; Hussain et al. 22, 21; Nadeem et al. 37). Such models are also currently used to analyze the flow of blood containing microorganisms and nanoparticles (Zhang etal. 62; Hussain et al. 22; Elogail and Mekheimer 18). Nanoparticles can be carriers of anticancer genes/drugs, biosensors, and cells that facilitate imaging. To analyze the flow of real blood vessels, it is necessary to use CFD simulations with the geometry of the vessel model based on in vivo magnetic resonance imaging (Yang et al. 58; Leach et al. 30) or computed tomography angiography (Zhu et al. 63; Aziz and Singh 7; Totorean etal. 53).

The CFD method is used to simulate blood flow in an abdominal aorta (AA) for atherosclerosis (Bonert et al. 10; Taylor et al. 51; Alimohammadi et al. 3), aortic stenosis (Alishahi et al. 4) and abdominal aortic aneurysms (Tanweer et al. 50; Dalman etal. 15; Kontopodis et al. 25; Scotti and Finol 46). Numerical simulations allow for the determination of the velocity and pressure profiles, as well as the distribution of a wall shear stress. An important aspect of the simulation of blood flow is the selection of an appropriate flow model. Depending on the size of the blood vessels, we get the Reynolds number of the order of 1 for small arterioles, and approximately 4000 in the largest artery (Ku 27; Lee et al. 31). For small values of the Reynolds number, or with simple well-built blood vessels, it is possible to use a laminar flow model. For large values of the Reynolds number, exceeding 2300, it is possible to use turbulent flow models. A turbulent flow can also occur at lower Reynolds numbers if there is a strong constriction in a blood vessel (Deshpande and Giddens 16) or if there are multiple stenoses (Lee et al. 31).

**Fig. 1:**
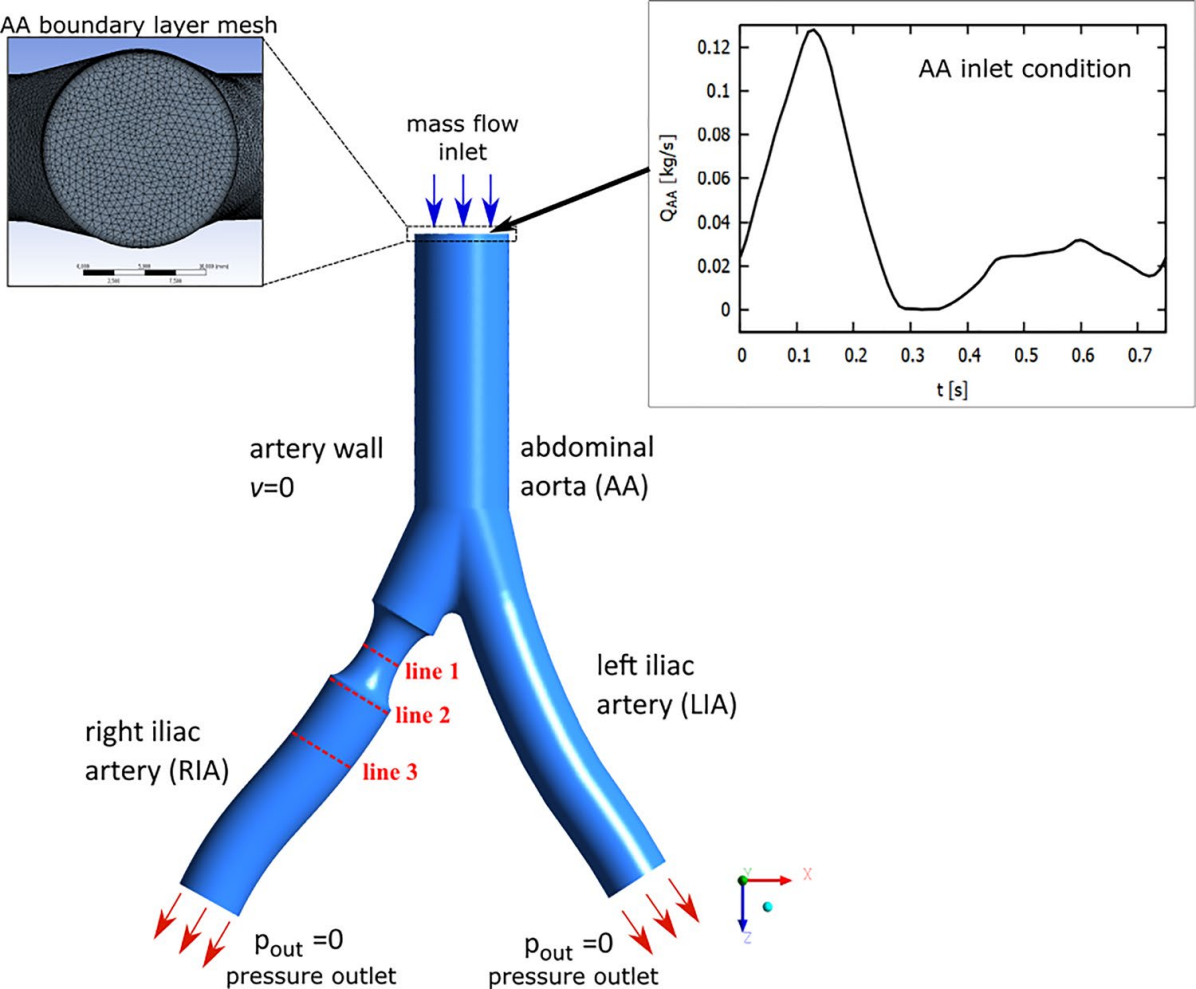
3D geometry of an abdominal aorta (AA) and iliac arteries (IA), with local 40% stenosis in the right iliac artery. The boundary conditions selected were: the mass flow at the inlet of AA, the constant zero gauge pressure at the outlet iliac arteries and the no-slip boundary condition with zero velocity for a blood-vessel interface. The inlet mesh grid is shown

Flow measurements in the abdominal aorta and common iliac artery of resting patients, when Reynolds numbers were in the range of 400-1100, did not show significant turbulence in blood flow (Stein et al. 48). In the case of high blood flow velocities and the presence of advanced atherosclerotic plaques in the abdominal aorta or iliac arteries, flow turbulence can occur. As Günenç etal. (20) shows that large atherosclerotic plaques occur in the abdominal aorta or common iliac arteries. In this paper, we consider pulsating blood flow in the abdominal aorta and common iliac arteries. We assume artery stenosis in one of the iliac arteries (see Fig. 1).

It is generally assumed that blood exhibits a non-Newtonian viscosity, so Non-Newtonian models such as Casson, Carreau-Yasuda, and Power-Law models are used to analyze the blood flow. On the other hand, in the case of large arteries with flows of high shear rate, it is possible to use the Newtonian model with constant viscosity value (Lynch et al. 34). Lynch et al. showed that the in-plane velocity and vorticity are larger in the Newtonian approximation but the wall shear stresses are larger in the non-Newtonian case. Newtonian and non-Newtonian approximations were used to analyze blood flow through the abdominal aorta (Bonert et al. 10; Alishahi et al. 4; Kumar et al. 29; Oliveira et al. 38; Stergiou et al. 49; Kotmakova et al.. 26; Zhan et al. 60; Tzirakis etal. 55).

The purpose of this work is to analyze the impact of selected flow models on the observed velocity distributions and the shear stress of the wall. The laminar, standard $k-\omega$, and standard $k-\epsilon$ models will be used for the analysis. Laminar models have been used quite often to model the flow in the bifurcated abdominal aorta (Taylor et al. 51; Alishahi et al. 4; Kotmakova et al.. 26). These models allow the use of Newtonian and non-Newtonian viscosities. Turbulence models (such as $k-\omega$ and $k-\epsilon$) have been used most commonly for a simple blood vessel with one (Ryval etal. 45; Varghese and Frankel 56; Parissis etal. 41; Banks and Bressloff 8; Kabir et al. 23; Teixeira et al. 52) or more (Lee et al. 31; Bernad et al. 9) arterial stenosis. The $k-\epsilon$ model was also used to analyze flow in the bifurcated abdominal aorta, but in this analysis only Newtonian viscosity was considered (Carvalho etal. 12). Parissis etal. (41) compared the numerical and experimental results for the flow through the by-pass region. The experimental setup represented an occluded artery with anastomosis. The authors showed that the $k-\omega$ model gives very comparable results compared to the experimental visualizations. The comparison of classic Reynolds-averaged Navier–Stokes (RANS) and Large Eddy Simulation (LES) models (Lopes et al. 33) also shows a good agreement of the $k-\omega$ model with the results of the LES models.

## Model

In this paper, we use the idealized rigid wall model of the abdominal aorta (AA) with two iliac arteries (IA) (see Fig. 1). The geometry is developed based on: actual diameter measurements of the AA and IA, position of the aortic bifurcation, and aortic bifurcation angle presented in Deswal etal. (17). The model assumes that the diameter of the inlet aorta is 16 mm, i.e. a value which is very close to the average value obtained in the mentioned paper. For both iliac arteries, in the bifurcation area, the diameter equal to 12 mm is assumed. In the geometry considered, the angle of aortic bifurcation equal to $49.37^\circ$ is used, with the aortic-iliac take-off angles $\alpha _R=29.62^\circ$, and $\alpha _R=19.75^\circ$ on the right and left sides, respectively. The lengths of the common iliac on the right and left sides are 58 and 50 mm. The length of the aortic bifurcation region is 18 mm. For the right iliac artery (RIA), we consider two cases: (i) healthy artery and (ii) unhealthy artery with local stenosis with 20% or 40% diameter reduction (36% or 64% area reduction, respectively). We always treat the left iliac artery (LIA) as healthy.

We performed the numerical analysis using the ANSYS Fluent 14.5 software. For blood, a tetrahedral mesh with 7 inflation layers in the boundary layer was used. We use a mesh element size equal to 0.6 mm. The choice of this value was preceded by the independent study of the model mesh. Sensitivity analysis is performed for transient simulation for the $k-\omega$ model and the 40% diameter reduction of the RIA. The table 1 presents the results for the maximum values of velocity ($v_{max}$) and *Z* direction wall shear stress ($WSS_{Z max}$) for three mesh element sizes. The results are obtained for a systolic peak equal to $t=0.12$ s. The results obtained show that a relative difference in speed for the Fine and Medium mesh is approximately 3%, while for the Medium and Coarse mesh the results are identical. In the case of *Z* direction wall shear stress, we obtain the relative difference of the order of 1% for Fine and Medium mesh, and of the order of 0.5% for Medium and Coarse mesh. Reducing the size of the mesh element does not significantly affect the results, so we used a Medium mesh for the calculations.

The mesh parameters are presented in Table 2. ANSYS software provides several tools to assess the quality of a mesh. Skewness is one of the basic measures of mesh quality. It determines how close to the ideal a face or cell is. The skewness values are in the range of 0-1, where 0 means perfect quality of the mesh structure and 1 means degenerate structure. For excellent mesh quality, this parameter must be in the range of $0 - 0.25$. The average skewness obtained for our geometry is approximately 0.192, which indicates very good mesh quality. The maximum values of skewness are 0.84−0.88, but they occur only for a few elements, so they are within the range of acceptable geometry. The second parameter, which describes the quality of a mesh, is the quality of the mesh element. It is a dimensionless quantity between 0 and 1, where 1 represents a perfectly regular element and 0 represents a degenerate one. The average value of 0.64 we obtained indicates good mesh quality.

**Table 1:** Mesh independent study

Mesh	Element size [mm]	Number of elements	$v_{max}$ [m/s]	$WSS_{Z max}$ [Pa]
Coarse	0.7	781,969	1.11	22.32
Medium	0.6	1,177,452	1.11	22.41
Fine	0.5	1,939,354	1.07	22.48

Another important parameter of the quality of a mesh, used especially for the $k-\omega$ and $k-\epsilon$ models, is $y^+$, which is a dimensionless parameter that represents the distance from the wall to the center of the first cell of the grid. This parameter allows us to assess whether the discretization of the boundary layer allows reproducing the velocity profile with sufficient accuracy. For the viscous sublayer, it is required that $y^+<5$. The ANSYS Fluent software allows evaluating this parameter for the $k-\omega$ and $k-\epsilon$ flow models. The values of $y^+$ we obtained, estimated for the highest flow velocity (systolic peak), fall within this range (see Table 2).

In the calculations, we used a velocity pulse with a period of $T=0.75$ s, which corresponds to a heart rate of 80 bpm. The computations were performed with a 0.01s time step. Since Transient Flow Simulations in Ansys Fluent package give inaccurate results in the first time steps, our simulations were performed for two cardiac cycles, and the results from the second cardiac cycle were used in the analysis. In our model, we used a time-dependent mass flow boundary condition for the inlet abdominal aorta. The profile of this flow was developed on the basis of the results of blood velocity presented by Alishahi et al. (4). The mass flow value at the input varies in the range between 0−0.128 kg/s (see Fig. 1). A similar maximum mass flow for the abdominal aorta was measured by Olufsen et al. (39); Amanuma et al. (6). Due to the specificity of Ansys Fluent simulation, we do not take into account reverse flow in the inlet, but reverse flow in the outlet will be possible. For the iliac arteries outlets, we assumed the constant zero gauge pressure boundary condition. For the walls of blood vessels, we applied a no-slip boundary condition with zero velocity for the fluid-solid interface. For the residuals of the continuity equation and the X, Y, and Z velocities, the convergence criteria were set to $10^{-4}$.

**Table 2:** Mesh parameters

	Healthy	Arterial stenosis	
		20%	40%
Elements	1,178,858	1,302,994	1,177,452
Nodes	313,832	354,946	317,562
Skewness			
Max	0.95	0.95	0.95
Average	0.198	0.203	0.201
Elements quality			
Average	0.68	0.67	0.68
$k-\omega$ model			
Max $y^+$	0.7	0.71	0.82
Average $y^+$	0.31	0.3	0.31
$k-\epsilon$ model			
Max $y^+$	0.74	0.76	0.97
Average $y^+$	0.38	0.37	0.38

Pulsating blood flow in large vessels, including the abdominal aorta, causes blood flow to be laminar at low speed most of the time. On the other hand, for the systolic peak, we observe high flow velocities that can cause turbulent flow. Narrowing of the vessel diameter caused by atherosclerotic plaques also causes flow disturbances. For these reasons, it is important to compare the results for laminar and turbulence models. With the given boundary conditions, we obtain the value of the average input velocity in the range of 0$-$0.6 m/s, which gives the Reynolds number $Re<4300$. This result is close to the value for the transitional flow. For higher input velocities, turbulent blood flow may be important, so we will use three models to analyze the flow: laminar flow model and two turbulence models such as standard $k-\omega$ and standard $k-\epsilon$ model (Kabir etal. 24). In our analysis, blood is treated as a Newtonian fluid with constant dynamical viscosity $\eta =0.0035 \text { Pa}\cdot \text {s}$ and a density $\rho =1060 \text { kg}/\text {m}^3$. So, for the abdominal aorta, the Womersley number is equal to $W_0=R\sqrt{2\pi \rho /\eta T}\approx 12.56$ i.e. it is within the range of $13\pm 1$ estimated in Ponzini et al. (43).

## Results

**Fig. 2:**
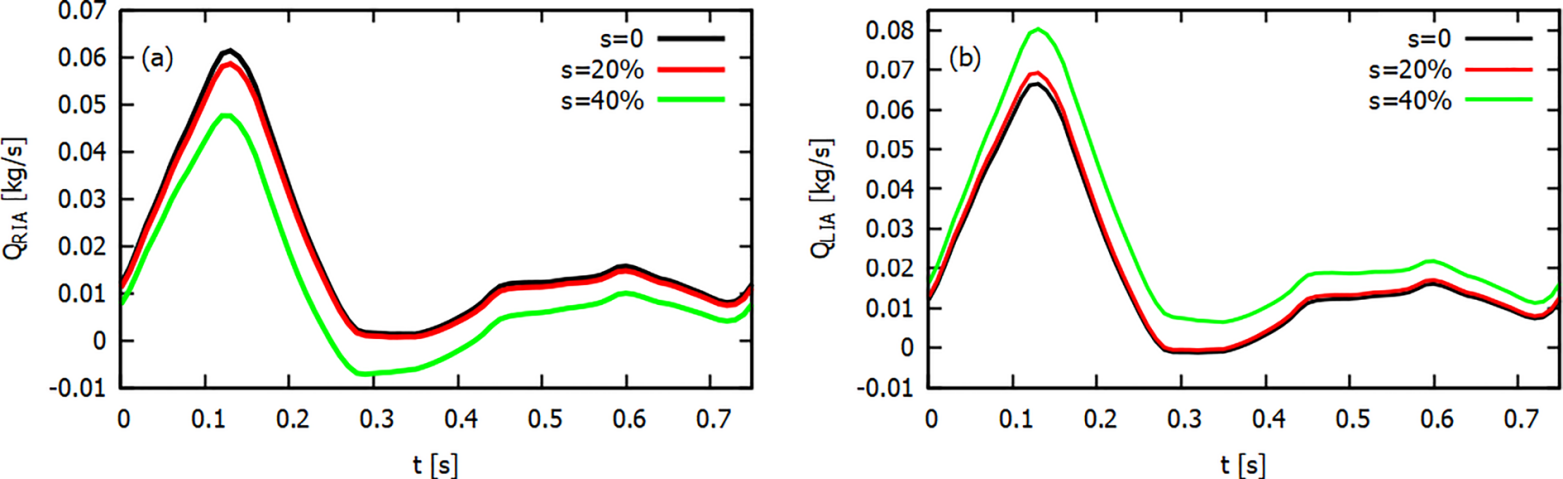
Mass flow waves at the outlet of right** a** and left** b** iliac artery obtained in the laminar flow model

**Fig. 3:**
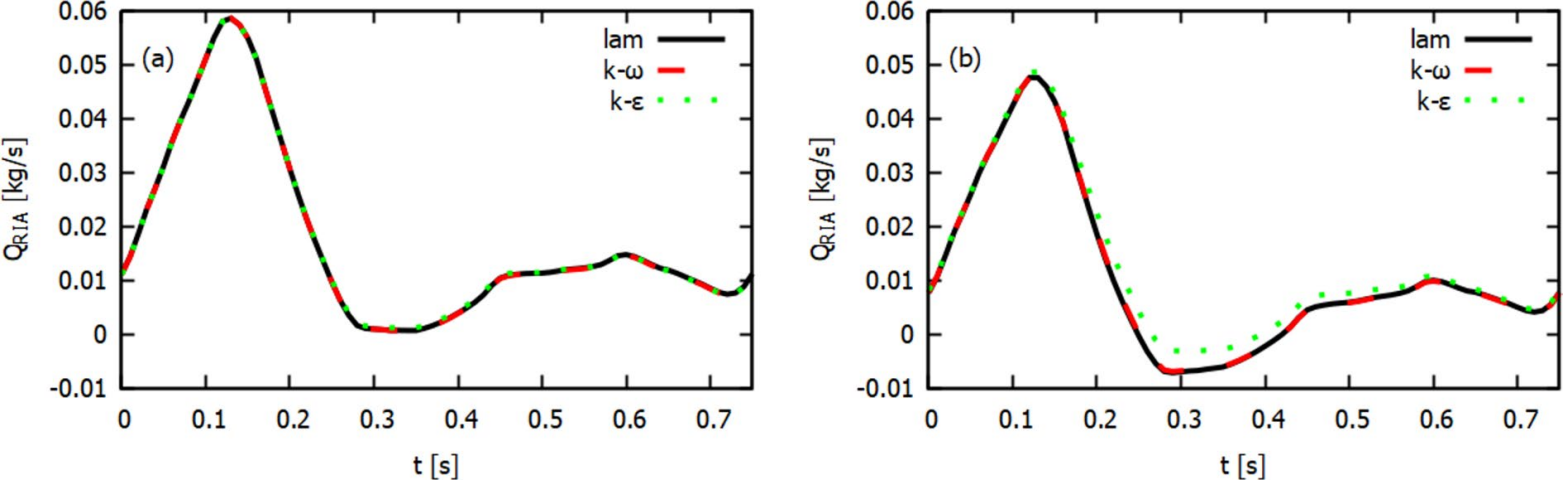
Mass flow waves at the outlet of right iliac artery obtained for 20% ** a** and 40% ** b** diameter reduction for three flow models

### Mass flow

In the first part of our analysis, we will focus on the numerical results of a mass flow at the outlet of the iliac arteries. In Fig. 2 we present mass flow waves at the outlet of the iliac arteries obtained for the laminar model. For both healthy arteries, the mass flow profiles are similar. In the case of an atherosclerotic lesion, we observe that there is a decrease in mass flow in the stenosis iliac artery (see Fig. 2(a)) and, for a strong narrowing (40%), a reverse blood flow occurs. For the left, healthy artery, the increase in artery stenosis in the RIA causes an increase in the mass flow of the left artery (see Fig. 2(b)). For the systolic peak and the 40% stenosis, the relative difference between a mass flow in LIA and RIA is of the order of 66%. The systolic peak of mass flow in the outlet of the arteries occurs at the same time as the peak at the AA inlet.

In Fig. 3 we present mass flow waves at the outlet of RIA for the diameter reduction 20% (a) and 40% (b) obtained for three flow models. For a smaller diameter reduction, it can be seen that the results for all three models are very similar. For a systolic peak, $t=0.12$s, the difference between a mass flow for $k-\epsilon$ and $k-\omega$ models is 0.06% only. At 40% diameter reduction one can see that for turbulent models, especially for the $k-\epsilon$ model, we obtain higher values of the mass flow than for the $k-\omega$ model. In this case, the difference is of the order of 1.9%. The results for the laminar and $k-\omega$ models are still very close.

**Fig. 4:**
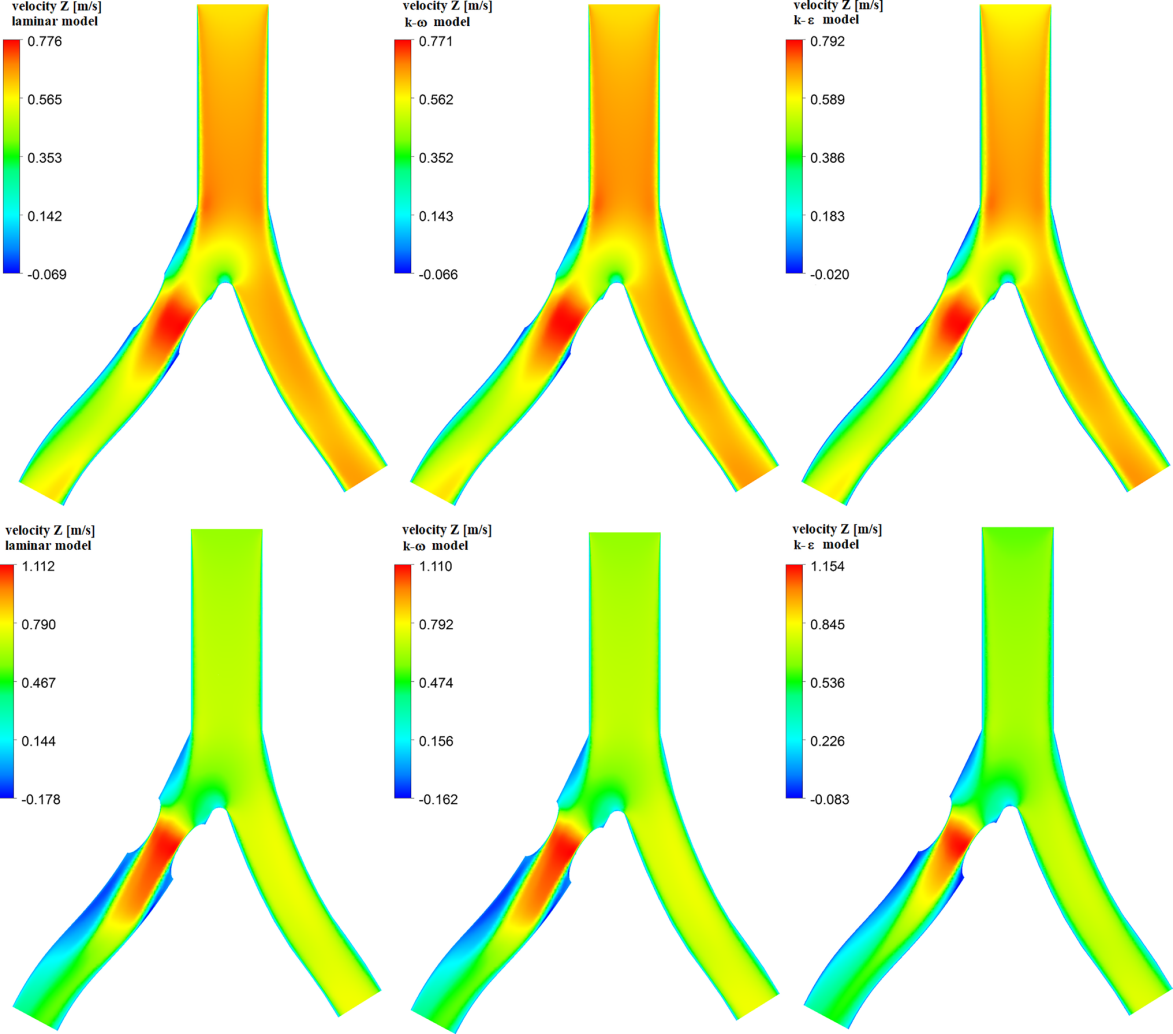
Z-direction velocity contour plots at XZ-plane ($y=0$) for 20% (top row) and 40% (bottom row) diameter reduction, for different flow models for a systolic peak

**Fig. 5:**
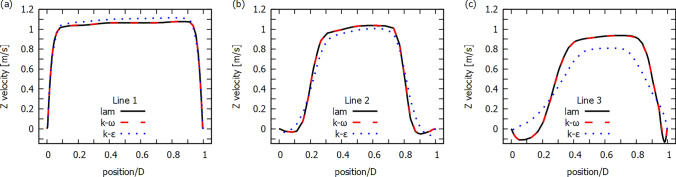
Z-direction velocity profiles for a systolic peak for different flow models, 40% diameter reduction and for line 1 (**a**), line 2 (**b**) and line 3 (**c**) marked in Fig. 1. Position is defined along a given line and relative to diameter (*D*) and assuming that 0 corresponds to left side and 1 corresponds to the right side (frontal view)

### Blood velocity

The calculations of a mass flow showed that for 40% diameter reduction, the choice of flow model used in the numerical analysis may be important. Now, we will check how the choice of the model we use affects the distribution of the blood flow velocity. In Fig. 4 we show the velocity distribution in the *z* direction (see Fig. 1) and for the systolic peak $t=0.12$ s. The top row of Fig. 4 is for 20% diameter reduction, while the bottom row is for 40% diameter reduction. Negative values correspond to reverse blood flow, that is, the areas where vortices form. For 20% diameter reduction, the velocity distribution for all three flow models is very similar. The maximum velocity values are almost the same, but there is a difference in the velocity for reverse blood flow. The results for laminar and $k-\omega$ models are similar, but for the $k-\epsilon$ model, we obtain lower values of a reverse flow velocity.

For 40% diameter reduction, in the area just behind the atherosclerotic lesion, there is a clear difference between the velocity distributions depending on the model used. As before, similar velocity profiles with a similar range of values are obtained for the laminar and $k-\omega$ models. For RIA, in the area behind the atherosclerotic lesion, a large region with negative values of velocity, corresponding to the reverse blood flow, is visible.

In Fig. 5 we present the velocity profiles for 40% diameter reduction and for three selected lines assuming that $y = 0$ (see Fig. 1). Line 1 is located at the narrowest point of the atherosclerotic lesion, line 2 is located just behind the atherosclerotic lesion and line 3 is located at a greater distance from the atherosclerotic lesion. The position of the measurement points is selected relative to the diameter of the artery cross-section for a given line ($D=$7.2 mm for line 1, $D=$11.87 mm for line 2 and $D=$11.81 mm for line 3) and assuming that 0 corresponds to the left side and 1 corresponds to the right side (frontal view). For all lines, the results for laminar and $k-\omega$ models are very similar.

For the $k-\epsilon$ model, the velocity profile is clearly different. The maximum velocity value in the RIA narrowing area (line 1) is higher than for other models, while the velocity value after the narrowing area drops faster (lines 2 and 3) than in the other two models. In addition, the velocity value for the reverse flow is smaller (line 2). Line 3 shows that for the laminar and $k-\omega$ model, the area of occurrence of the reverse blood flow is higher than for the $k-\epsilon$ model. A similar difference between $k-\omega$ and $k-\epsilon$ models was shown by Varghese and Frankel (56) and (Banks and Bressloff 8) for simple blood vessel with arterial stenosis. The results for $k-\omega$ model obtained by Varghese and Frankel (56) were similar to the experimental data obtained from laser Doppler velocimetry (Ahmed and Giddens 1). For the flow through LIA, the velocity values in the middle of a profile are very similar for all three models.

**Fig. 6:**
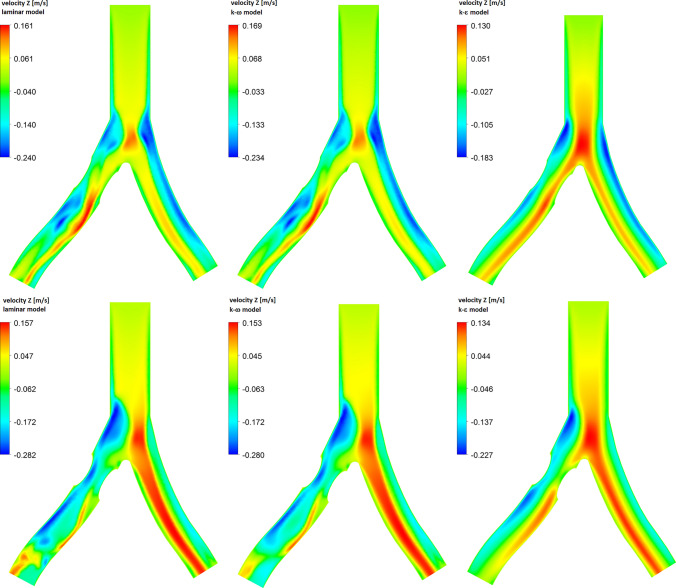
Z-direction velocity contour plots at XZ-plane ($y=0$) for 20% (top row) and 40% (bottom row) diameter reduction, for different flow models and at the end of the systolic phase ($t=0.29s$)

Differences between the models used can also be seen at low flow velocities, for which we obtain low values of the Reynolds number. It is accepted in the literature that for the low Reynolds number the $k-\omega$ model gives more accurate results than the $k-\epsilon$ model. In Fig. 6 we show the Z-direction velocity contour plots at the end of systolic phase ($t=0.29s$). In this case, for narrowed RIA and in the bifurcation area we observe the reverse blood flow, illustrated by negative values of the Z-direction velocity. The results for laminar and $k-\omega$ model are very similar. For 40% diameter reduction (bottom row of Fig. 6) in RIA there is a clear flow disturbance in these two models. For the $k-\epsilon$ model the flow is less disturbed. The range of flow velocity values is also smaller.

As we will see, the negative velocity distribution in the Z-direction will be closely related to the negative Z-direction wall shear stress in the area behind the atherosclerotic lesion. This behavior will be presented in the next chapter.

**Fig. 7:**
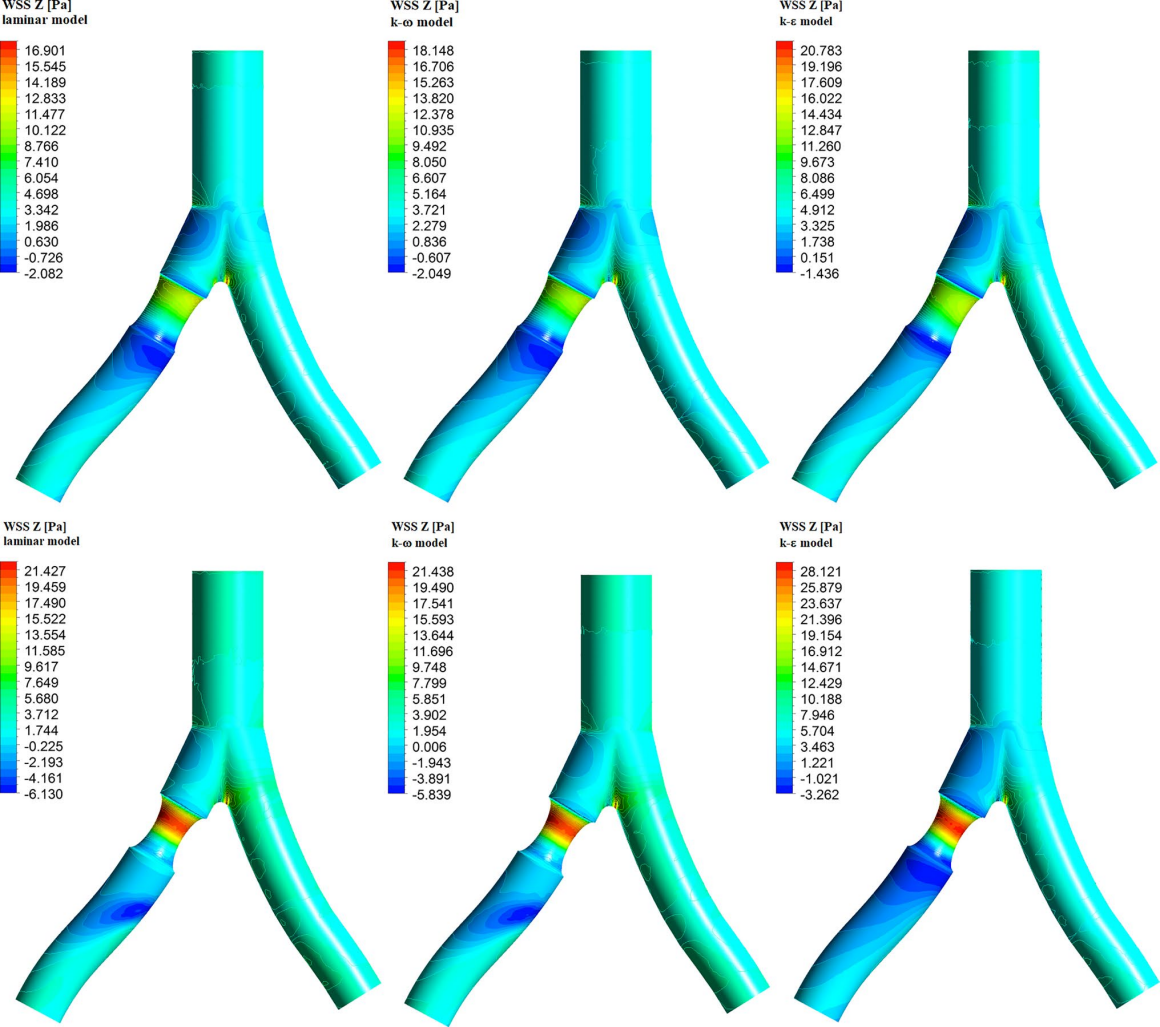
Z-direction wall shear stress for 20% (top row) and 40% (bottom row) diameter reduction, for different flow models and for the systolic peak

### Wall shear stress

Aortic areas characterized by low wall shear stresses (WSS) and high oscillatory shear index (OSI) are prone to the formation of atherosclerosis (Ku et al. 28; Zarins et al. 59; Peiffer et al. 42; Malek et al. 35; Zhang et al. 61). Low WSS and high OSI are often observed near the aortic bifurcation (in the outer walls) or in the inner curvatures of the vessels. These areas are characterized by slow blood flow and by the appearance of reverse blood flow. This effect also occurs behind the atherosclerotic lesion, which narrows the diameter of the blood vessel. Strong flow oscillations and relatively long time the cells stay in the artery allow the penetration of cells involved in the atherosclerotic process into arterial walls. However, high WSS values occur on the inner walls of the bifurcation and on the top of an atherosclerotic plaque. One of the concepts considered in the literature concerning this problem assumes that high WSS can be related to the occurrence of high-risk plaques (Eshtehardi et al. 19; Brown et al. 11). Especially for WSS values that exceed plaque strength, plaque rupture can occur.

**Table 3:** Wall shear stress for different flow models

		Healthy	Arterial stenosis	
			20%	40%
Maximum WSS [Pa]				
laminar		22.67	24.97	32.04
$k-\omega$		23.56	25.74	32.49
$k-\epsilon$		26.43	29.02	36.75
Z-direction WSS [Pa] for $t=0.12$s				
laminar	Max	16.97	17.58	22.41
	Min	$-$0.93	$-$2.08	$-$6.13
$k-\omega$	Max	18.23	18.87	22.41
	Min	$-$0.94	$-$2.05	$-$5.84
$k-\epsilon$	Max	20.68	21.58	29.24
	Min	$-$0.07	$-$1.44	$-$3.26

The value of wall shear stress depends on the flow rate and the diameter of the vessel, so the maximum values of WSS will be obtained for systolic peak and for 40% diameter reduction of RIA. In Table 3 we present the results for the maximum values of WSS and for Z-direction values of WSS for the three models used by us. The maximum value of WSS depends on the flow model used. For the laminar and $k-\omega$ models we obtain very similar values (32.04 Pa and 32.49 Pa), while for the $k-\epsilon$ model the maximum value of WSS is equal to 36.75 Pa, that is, it is 15% higher than in the laminar model. For the 20% diameter reduction of RIA we obtain 24.97 Pa, 25.74 Pa, and 29.02 Pa, respectively. For a healthy artery, the maximum values of WSS are obtained for the inner walls of the bifurcation and are 22.67 Pa, 23.56 Pa and 26.43 Pa, respectively.

In Fig. 7 we present the wall shear stress for the Z-direction and for the systolic peak, $t=0.12$ s. Negative values of WSS are related to reverse blood flow. The results for the laminar and $k-\omega$ models are similar, while for the $k-\epsilon$ model we have higher maximum values of WSS and lower values of WSS for reverse flow. In this model, a negative WSS occurs just behind the atherosclerotic lesion, and for the two previous models the most negative value of the WSS can be observed at some distance from a plaque.

**Fig. 8:**
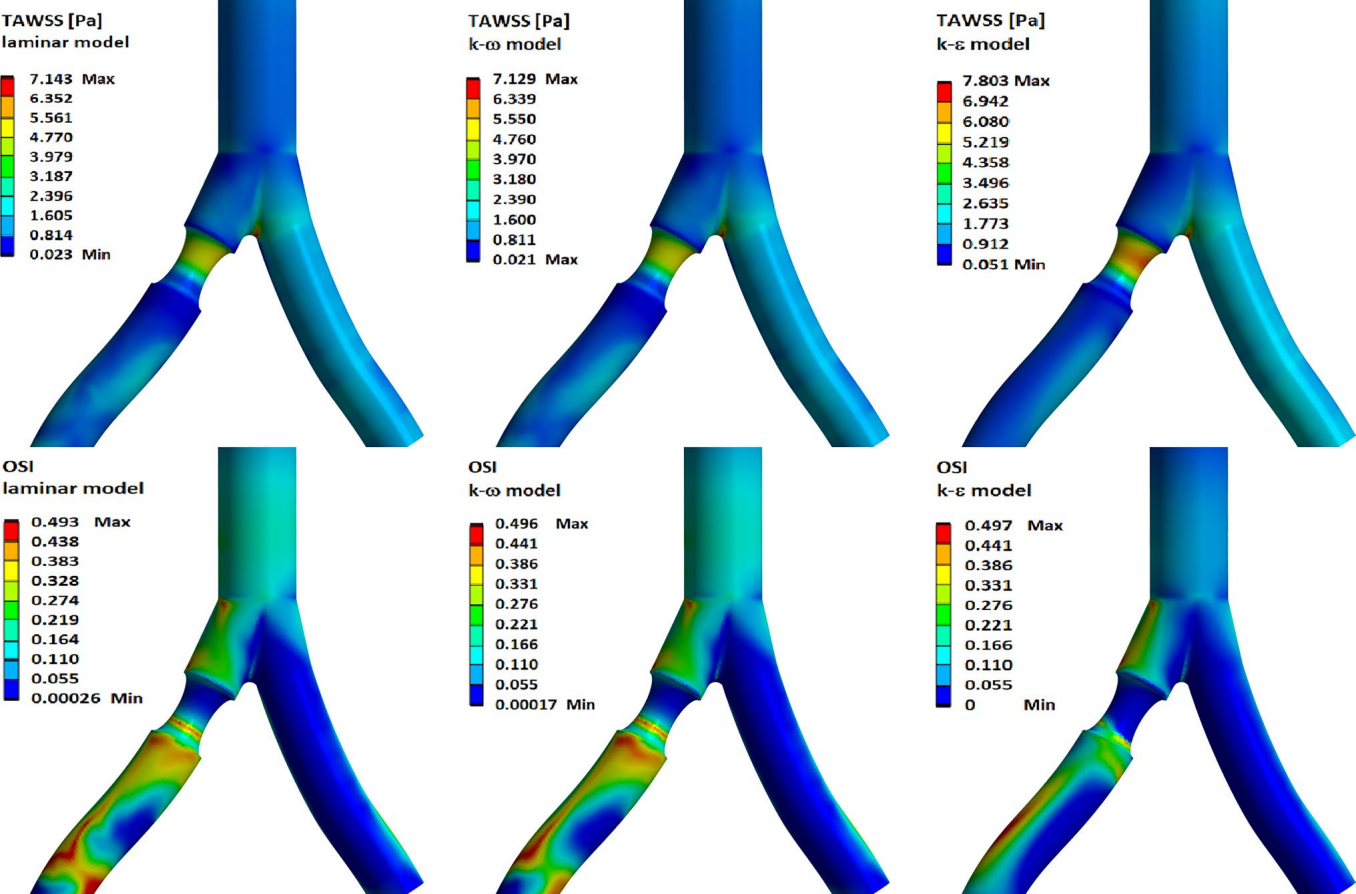
The time-averaged wall shear stress (top row) and oscillatory shear index (bottom row) for different flow models. The computations were made for 40% diameter reduction of RIA

In Fig. 8 we present distributions of the time-averaged wall shear stress (TAWSS) and oscillatory shear index (OSI) for different flow models and for 40% diameter reduction of RIA. The time-averaged wall shear stress was calculated by use of the following formula (Kotmakova et al. 26):

1 $$\begin{aligned} TAWSS=\frac{1}{T} \int _0^T{\left| {\tau }_w\right| dt}, \end{aligned}$$

where ${\tau }_w$ is the vector of wall shear stress and *T* is the pulsation period. The oscillatory shear index is given by Taylor et al. (51):

2 $$\begin{aligned} OSI=\frac{1}{2}\left( 1-\frac{1/T \left| \int _0^T{\mathbf {\tau }_w dt}\right| }{ TAWSS}\right) . \end{aligned}$$

For healthy iliac arteries, the minimum value of TAWSS is obtained behind the aortic bifurcation region on the outer walls of the arteries. In this region, we also observe the maximum values of OSI. High values of TAWSS and low values of OSI are obtained in the inner wall of the aortic bifurcation.

In the case of an atherosclerotic lesion, high TAWSS values are observed on the top of the atherosclerotic plaque, while low TAWSS values and high OSI values are obtained just behind the plaque (see Fig. 8). For the $k-\epsilon$ model, the maximum value of TAWSS is higher than the corresponding value for the $k-\omega$ and laminar models. On the other hand, high values of OSI are obtained for a larger area for the laminar and $k-\omega$ models. This is because in the laminar and $k-\omega$ models we obtain stronger reverse flow than in the $k-\epsilon$ model (see Fig. 4).

The above distributions of TAWSS and OSI values were obtained based on calculations performed in the Wolfram Mathematica 9.0.

The following algorithm is used for calculations and presentation of results:

Step 1: Using the results obtained in Ansys Fluent Solution, for each time step, the wall shear stress values for the X, Y, and Z directions) occurring for each point of the mesh on the surface of the artery wall were retrieved.

Step 2: Based on the data obtained in the previous step, the TAWSS and OSI values were calculated in the Wolfram Mathematica 9.0 for each point of the mesh using Eqs. (1) and (2). The obtained TAWSS and OSI values should be saved to a file along with information about the location of the point of the mesh.

Step 3: Visualization of TAWSS and OSI values can be implemented in the ANSYS package using the External Data component, into which we import the previously obtained file, and ANSYS Static Structural software (imported load). The mesh size must be selected in such a way that it reflects the distribution of the obtained TAWSS and OSI values as accurately as possible.

### Results for real abdominal aorta

**Fig. 9:**
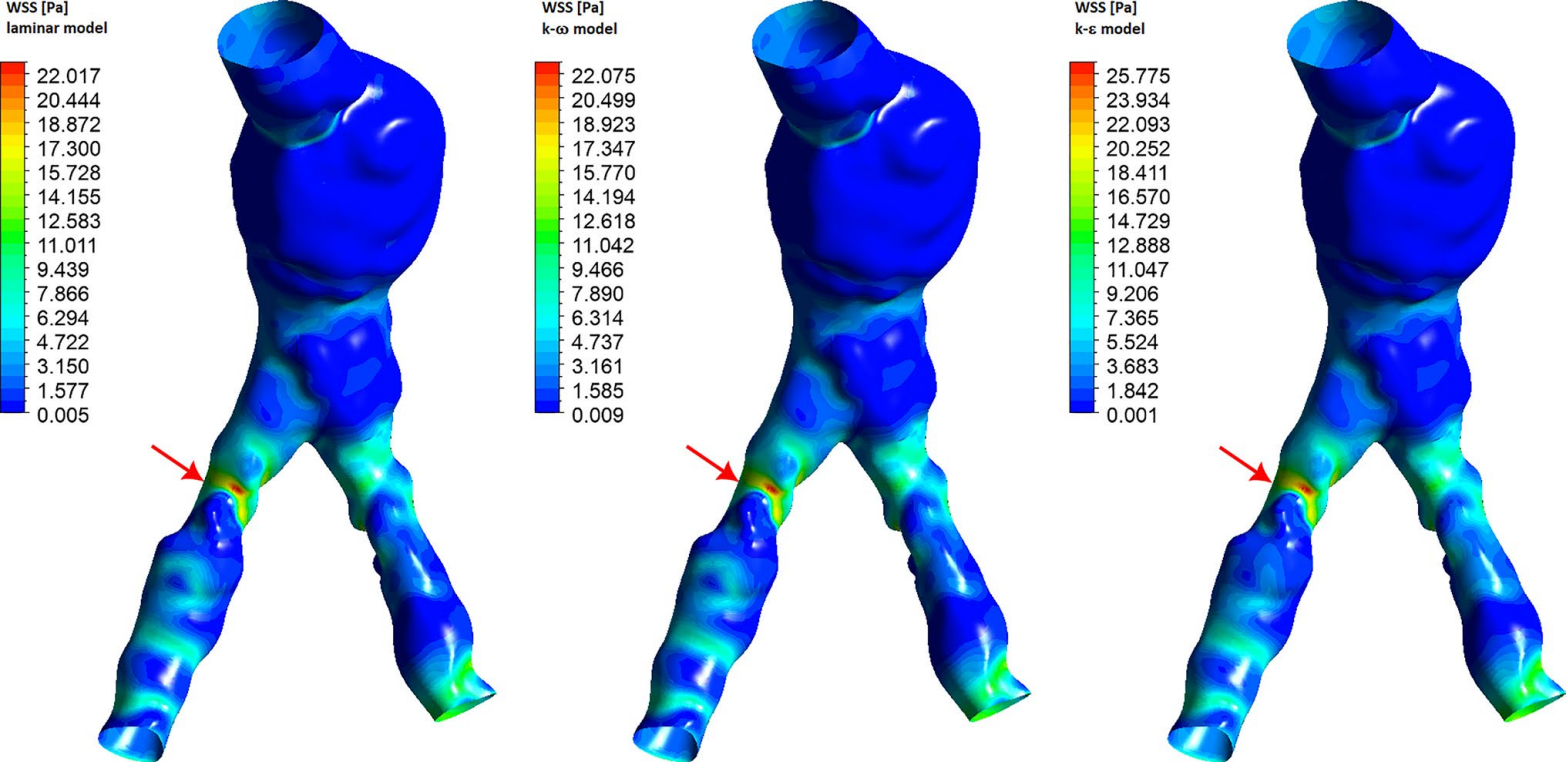
Wall shear stress for real abdominal aorta. The presented results correspond to the systolic peak ($t=0.12s$)

**Fig. 10:**
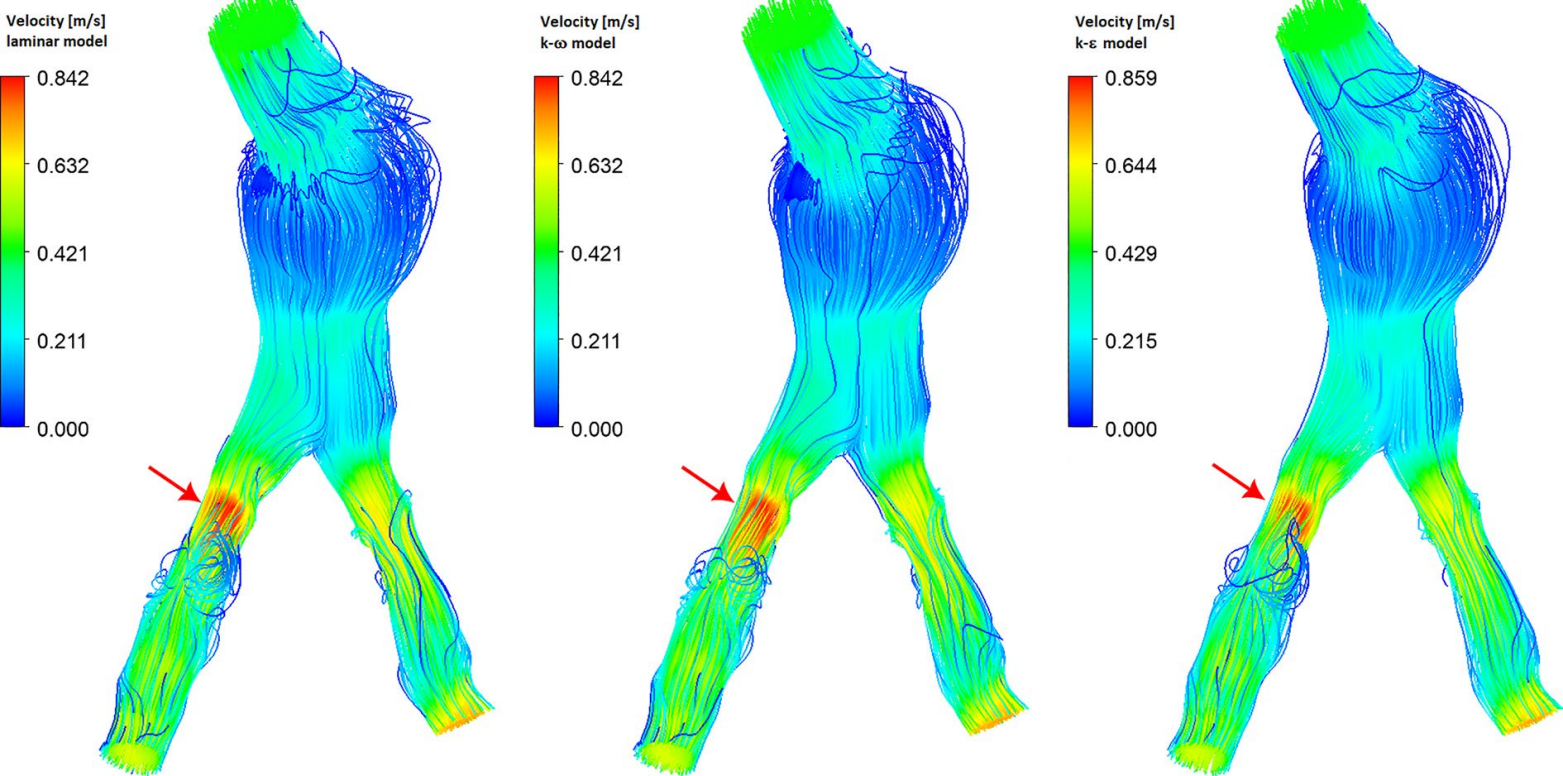
Velocity streamline for real abdominal aorta. The presented results correspond to the systolic peak ($t=0.12s$)

In the previous section, we presented the results obtained for an idealized model of the abdominal aorta. Currently, we will use real geometry for analysis. 3D geometry was reconstructed from computed tomography (CT) images using 3D Slicer software. The geometry obtained shows an abdominal aortic aneurysm, calcification of the aorta and common iliac arteries, and local narrowing of the iliac arteries. The surface of the abdominal artery inlet is 2.77 cm$^2$, while for RIA and LIA outlets we have surfaces equal to 1.41 cm$^2$ and 0.91 cm$^2$, respectively. Due to severe pathological changes, the shape of AA inlet and IA outlets is very irregular. The aortic bifurcation angle between RIA and LIA is about 45$^\circ$. Pathological changes caused severe local narrowing of iliac arteries. In Figs. 9, 10 and 14 the red arrow shows the place of severe RIA stenosis, where the cross-sectional area of the artery is 48% smaller compared to the cross-sectional area of the artery behind the stenosis. Such a severe narrowing of the RIA may cause significant disruption of blood flow. Additionally, local dilatation of the aorta can be observed in the area of aortic bifurcation. As in the previous calculations, in the analysis we used the time-dependent mass flow boundary condition for the inlet abdominal aorta, where the mass flow rate profile is presented in Fig. 1. For the iliac arteries outlets the constant zero gauge pressure boundary condition was applied. For the walls of blood vessels, we applied a no-slip boundary condition with zero velocity for the fluid-solid interface. The transient state analysis was performed for two cardiac cycles and the results of the second cycle were selected for presentation. A tetrahedral mesh with 7 inflation layers was used for the analysis, divided into 971,083 elements and 327,721 nodes with 0.58 average elements quality and 0.219 average skewness.

The wall shear stress distribution in the real model is presented in Fig. 9 for the systolic peak ($t=0.12s$). As for the idealized model, we find that the WSS for laminar and $k-\omega$ models are very similar, while the results for the $k-\epsilon$ model are clearly higher. The maximum WSS is obtained at the stenosis of one of the iliac arteries. The maximum value of WSS for laminar and $k-\omega$ models is equal to 22.8 [Pa], while for the $k-\epsilon$ model it is equal to 26.7 [Pa]. This value is approximately 17% higher than in other models. In the area of the aortic aneurysm, the WSS value is approximately 1 [Pa].

In Fig. 10 we present the velocity streamline for the real abdominal aorta. In the narrowing area, the highest velocity value is obtained for the $k-\epsilon$ model, while behind the narrowing, the velocity value drops faster for this model than for other models. A similar relationship is presented in Fig. 4. The obtained streamlines also show flow disturbances in the area of the aortic aneurysm and in the region behind the Iliac artery stenosis. The largest disturbances are obtained for the $k-\omega$ model and the smallest disturbances for the $k-\epsilon$ model.

**Fig. 11:**
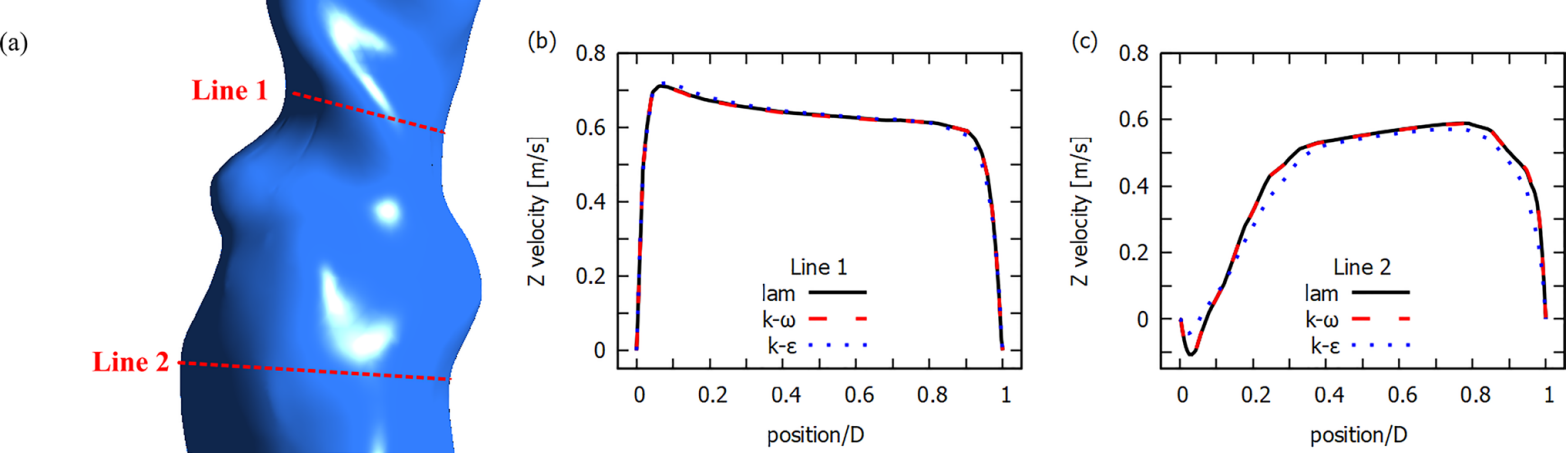
Z-direction velocity profiles for different flow models in the area of strong RIA stenosis (see red arrows in Fig. 9) and below the RIA stenosis. Panel (**a**) shows the location of both lines, Line 1 - panel (**b**), and Line 2 - panel (**c**). The presented results correspond to the systolic peak ($t=0.12s$)

In Fig. 11 we present Z-direction velocity profiles for different flow models. Line 1 is selected for the area of strong RIA stenosis while Line 2 is selected for the area below the RIA stenosis. For Line 1 we obtain very similar velocity values, while for Line 2 laminar and $k-\omega$ models show higher velocity values than the $k-\epsilon$ model. Also for the reverse blood flow the speed value is higher than for the $k-\epsilon$ model. These results are very similar to those for idealized geometry (see Fig. 5).

**Fig. 12:**
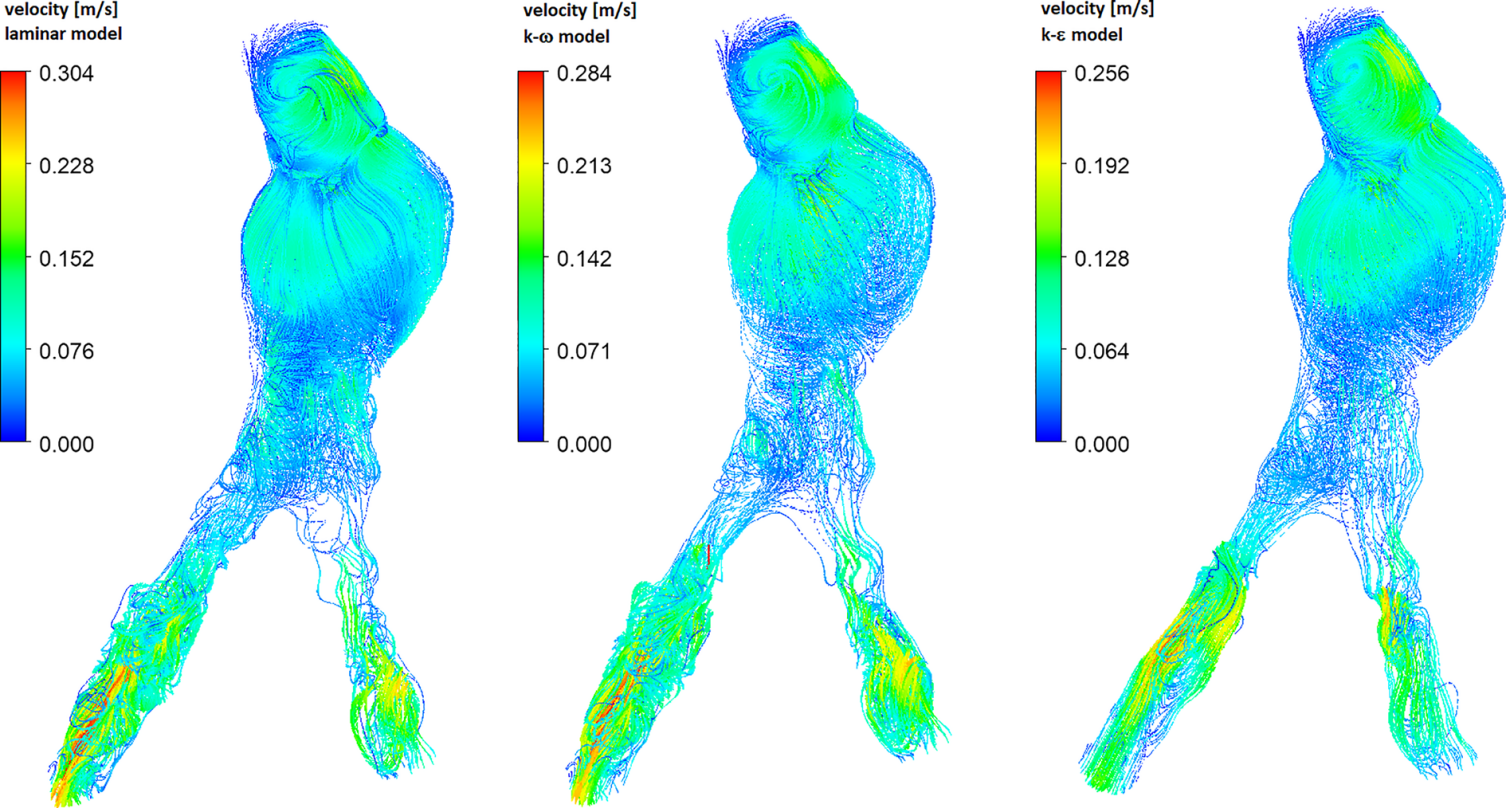
Velocity streamline for real abdominal aorta at the end of systolic phase ($t=0.29s$)

**Fig. 13:**
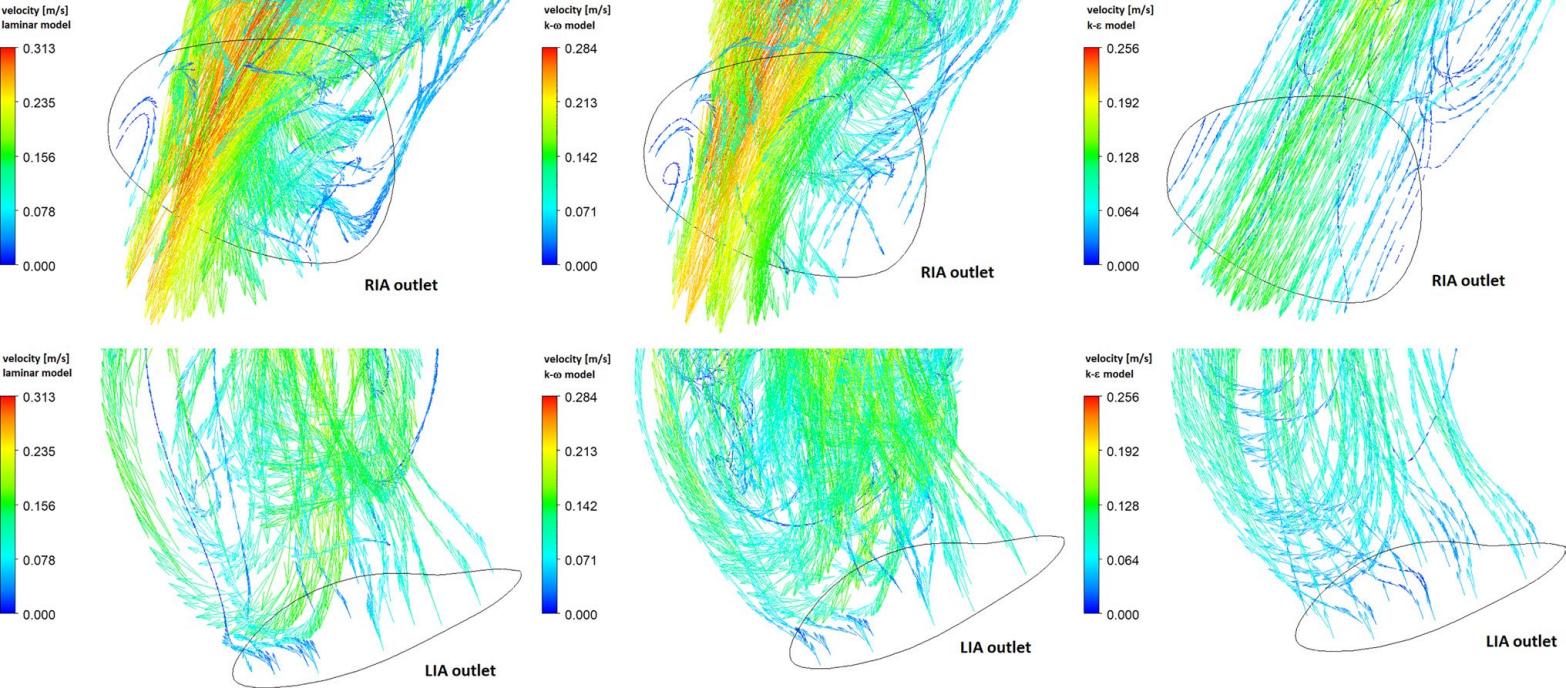
Vectors of velocity for the outlet of RIA (top row) and LIA (bottom row) obtained at the end of the systolic phase ($t=0.29s$)

In Figs. 12 and 13 we present velocity distribution at the end of the systolic phase ($t=0.29s$), i.e. for the case of low flow velocities. Figure 12 shows velocity streamlines for three flow models. Due to the specific geometry of the aorta, especially the occurrence of an abdominal aortic aneurysm, blood flow is realized through the RIA outlet. For iliac artery outlets, we observe the occurrence of the reverse blood flow (see Fig. 13). Due to the much smaller surface of the LIA outlet in comparison to the RIA outlet, the strongest reverse blood flow occurs in the LIA outlet (see the bottom row in Fig. 13). The total mass flow rate for the LIA outlet is negative for all three models. For the RIA outlet, which has a larger surface area than the LIA outlet, we observe forward and reverse blood flow (see the top row in Fig. 13) which results in an effectively positive mass flow rate of blood. Local narrowing of the RIA (marked with an arrow in the previous figures) causes flow disturbances to occur. For the laminar and $k-\omega$ models, the flow is more disturbed than for the $k-\epsilon$ model.

**Fig. 14:**
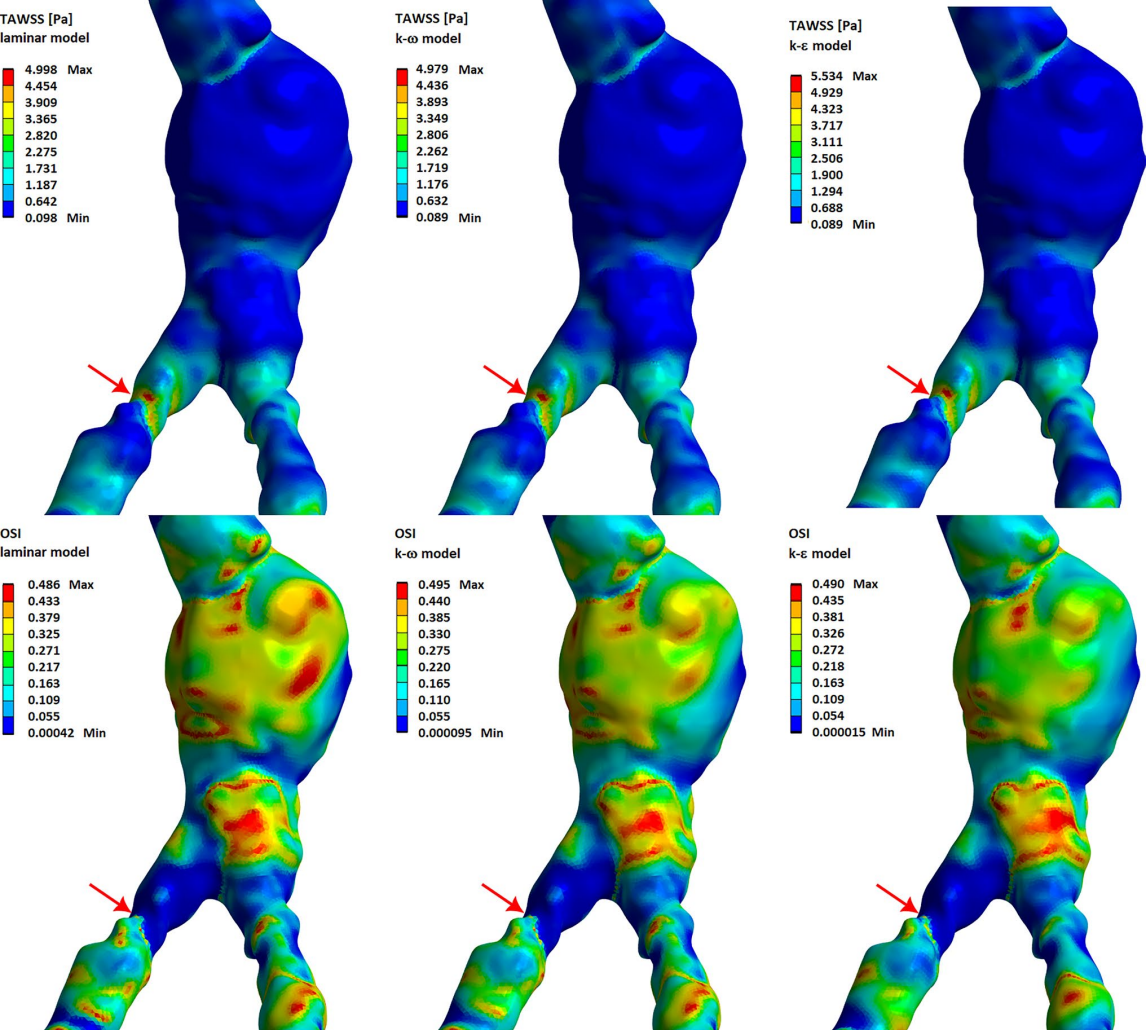
The time-averaged wall shear stress (top row) and oscillatory shear index (bottom row) for real abdominal aorta

In Fig. 14 we present the time-averaged wall shear stress (top row) and the oscillatory shear index (bottom row) for a real abdominal aorta. The results obtained show that the maximum TAWSS is in the area of iliac artery stenosis. However, very low values are obtained for aortic aneurysm and below iliac artery stenosis. In these two areas, strong velocity turbulences were obtained, so as a result we obtain high OSI values. A high value of OSI is also visible in the area of dilatation of aortic bifurcation. The simultaneous occurrence of AA dilatation, narrowing of the iliac arteries, and local calcification of the aorta and common iliac arteries greatly disturbed blood flow and may be a risk factor for the patient’s health.

### Limitations

The results we present have some limitations. We made the rigid wall assumption for both the idealized and real geometry. The use of an elastic wall should result in slightly lower values of the velocity and wall shear stress, but it would probably not affect the fact that the $k-\epsilon$ model gives higher values of these parameters.

The second limitation was the use of the inlet mass flow based on the data from the literature (Alishahi et al. 4). A better solution would be to use patient-specific flow data closely related to the real geometry used. However, this would require the use of a very accurate measurement method, e.g. used by Alimohammadi et al. (2).

Another limitation was that for inlet mass flow we assumed no reverse flow for the diastolic phase of the cardiac cycle. However, reverse mass flow can be observed for the outlet boundaries. Figures 2 and 3 show that with a strong stenosis of the RIA we obtain reverse mass flow in this artery.

## Conclusions

If an artery is narrowed, there are several surgical techniques to remove the stenosis. One of the most frequently used is balloon angioplasty and stent placement. The disadvantage of this procedure is the possibility of repeated restenosis. It is also possible to perform stenosis bypass. In this case, the parameters of the anastomosis should be carefully selected because subendothelial hyperplasia or thrombosis may occur in the area of anastomosis (Parissis etal. 41). The choice of treatment method by clinical doctors may be preceded by performing numerical simulations, but the simulation assumptions must be correctly selected, especially the flow model that most accurately illustrates the actual flow should be well selected.

Our goal was to compare blood flow parameters through a stenotic artery for laminar, $k-\omega$ and $k-\epsilon$ models. Analysis of blood flow through real blood vessels requires the use of geometry which is similar to geometry obtained in magnetic resonance or computed tomography images, which means that CFD simulation should be used for the analysis. We used two geometries in our analysis: the abdominal aorta and the iliac arteries. One of the geometries used was the idealized rigid wall model of the abdominal aorta and two iliac arteries. AA and IA sizes were selected based on data from the literature (Deswal etal. 17). To analyze the effect of IA stenosis on blood flow parameters, we introduced two degrees of RIA stenosis. The second geometry was reconstructed from CT images. Severe pathological changes are visible in the geometry obtained (abdominal aortic aneurysm, calcification of the aorta and common iliac arteries, and local stenosis of the arteries). In the case of large vessels (e.g. aorta, common iliac arteries), at higher flow velocities, turbulent flows may occur, especially in narrowing areas of large vessels. The choice of turbulence models (such as $k-\omega$ and $k-\epsilon$) affects the results obtained for the distribution of velocity and wall shear stress. In the current study, we compare the results for three flow models: the laminar model, the standard $k-\omega$ model, and the standard $k-\epsilon$ model. For idealized geometry, we performed CFD simulations of a healthy and stenotic right iliac artery. We compared the results for mass flow distributions, velocity, and wall shear stress for different flow models with an idealized model. The results obtained show that the laminar and $k-\omega$ models give very similar results, both for the velocity distribution and for the wall shear stress. The $k-\epsilon$ model gives higher values of velocity and WSS for the narrowed artery. On the other hand, the laminar and $k-\omega$ models are characterized by larger areas of occurrence of a reverse flow in the area behind the narrowing, showing the presence of a broader range of high velocity values in the middle of the stream and the appearance of a reverse flow near the boundary layers. In these models, the negative value of WSS occurs in a larger area than for the $k-\epsilon$ model.

The choice of flow model affects the values of time-averaged wall shear stress and oscillatory shear index. For a flow described by the $k-\epsilon$ model, we observe that the TAWSS values are 10% higher than in the case of the other models considered. Higher TAWSS values for $k-\epsilon$ model are related to higher flow rates obtained in this model. The results for the velocity profile also showed that in the laminar and $k-\omega$ models we obtain stronger flow turbulence corresponding to the reverse blood flow. This effect causes an increase in the OSI value. As a result, in the area behind the atherosclerotic lesion, much larger areas can be obtained with higher OSI values for the laminar and $k-\omega$ models than for the $k-\epsilon$ model.

The $y^+<2$ values obtained in our model are typical for the viscous sublayer. For such a range of $y^+$, the $k-\omega$ model is preferred. The obtained results show that in the case of severe stenosis of the right iliac artery below the stenosis area, one can observe stronger curvature of the velocity streamline, resulting in reverse flow in the boundary layer. This effect is stronger for the laminar and $k-\omega$ models. The $k-\epsilon$ model describes the flow less well near the vessel walls. Similar results were obtained by Parissis etal. (41).

Calculations performed for real geometry also show that the $k-\epsilon$ model gives higher velocity and wall shear stress values than the laminar and $k-\omega$ models. As for the idealized model, in real geometry we observe stronger flow velocity turbulence in the $k-\omega$ model than in the $k-\epsilon$ model. This affects the results of the oscillatory shear index, especially in the area of AA dilation and below the local IA stenosis. In summary, the comparison of $k-\omega$ and $k-\epsilon$ models shows that the $k-\epsilon$ model overestimates the values of both velocity and WSS. For the flow conditions that prevail in large vessels of the circulatory system, it is better to choose the $k-\omega$ model. Additionally, it should be noted that the results obtained by us show that the laminar and $k-\omega$ models give very similar results. Our results and the results reported in the literature (see e.g. Parissis etal. 41; Banks and Bressloff 8; Lopes et al. 33 indicate that $k-\omega$ is the most suitable for analyzing blood flow in large blood vessels. The laminar model also gives good results. These two models are also often used to describe blood flow in the coronary arteries (see review article Carvalho et al. 13).

## Data Availability

Data available on request from the corresponding author.
